# Medidas do Manguito Periférico para Estimativa da Pressão Aórtica Média

**DOI:** 10.36660/abc.20250543

**Published:** 2025-10-14

**Authors:** Weimar Kunz Sebba Barroso, Sayuri Inuzuka

**Affiliations:** 1 Universidade Federal de Goiás Faculdade de Medicina Unidade de Hipertensão Arterial Goiânia GO Brasil Unidade de Hipertensão Arterial - Faculdade de Medicina - Universidade Federal de Goiás, Goiânia, GO – Brasil

**Keywords:** Pressão Arterial, Medições

Por mais de um século, o método simples, realizado em consultório, de medir a pressão arterial na parte superior do braço com um esfigmomanômetro permaneceu o padrão estabelecido para sua medição e tratamento. De acordo com os princípios hemodinâmicos, a função primária do sistema cardiovascular é garantir a perfusão contínua de órgãos e tecidos vitais.^1^ A função cardíaca e a dinâmica vascular geram a força conhecida como pressão arterial.^1^ A pressão aórtica descreve a força dinâmica e pulsátil exercida dentro da maior artéria do corpo, refletindo a interação batimento a batimento entre o coração e a árvore arterial.^1^ Em contraste, a pressão arterial média (PAM) representa a pressão média temporal que serve como força motriz constante para o fluxo sanguíneo sistêmico.^2^ Uma compreensão abrangente de suas principais distinções, mecanismos fisiológicos subjacentes e significado clínico é crucial para o diagnóstico e tratamento adequados de um amplo espectro de doenças cardiovasculares.

A PAM é a pressão média que garante um fluxo constante e contínuo de sangue para os tecidos e órgãos do seu corpo.^2^ Essa perfusão constante é essencial para fornecer o oxigênio necessário para que as células funcionem adequadamente.^2^ A preocupação clínica é que para perfundir órgãos vitais, há uma exigência de uma PAM mínima de 60 mmHg.^3^ Se a PAM cair abaixo de 60 mmHg por um período prolongado, pode resultar em manifestações como isquemia e infarto.^3^ Existem várias fórmulas para o cálculo da PAM, e a fórmula de Gauer foi a primeira sugerida.^2^


PAM=PD+1/3(PS-PD)ou PAM=PD+1/3(PP)

Onde PD é a pressão arterial diastólica, PS é a pressão arterial sistólica e PP é a pressão de pulso. A pressão aórtica é uma forma de onda pulsante dinâmica que fornece informações sobre a qualidade de cada ciclo cardíaco, refletindo a força de contração, a integridade da válvula aórtica e as propriedades da árvore arterial.^1^ A PAM é um valor calculado em estado estacionário que resume o resultado médio desses ciclos, representando a força motriz da perfusão sistêmica.^4^

Os autores do artigo^5^ sugerem uma fórmula melhor para o cálculo da PAM (PAM=PD+[0.33+(0.16−0.00107×PP)+(−0,06+0,000773×FC)]×PP). Esta fórmula utiliza dados obtidos por um esfigmomanômetro convencional. Os dados obtidos a partir desta fórmula foram comparados com a pressão intra-aórtica medida por angiografia coronária. Ao longo dos anos, esforços significativos têm sido dedicados ao desenvolvimento de tecnologias que estimam a forma de onda da pressão central de maneira não invasiva a partir de um local de medição periférico.^6^ A tonometria de aplanação é a técnica mais amplamente validada para avaliar de maneira não invasiva a forma de onda da pressão central, e os avanços tecnológicos mais recentes integraram a análise da forma de onda em dispositivos oscilométricos de pressão arterial por meio de algoritmos específicos do dispositivo que são usados para derivar e estimar a forma de onda da pressão aórtica central a partir da forma de onda braquial.^6^

O valor preditivo da pressão central tem sido amplamente investigado em coortes com um grande número de pacientes, a maioria deles utilizando técnicas baseadas em tonometria, que relataram que a pressão central foi independente de eventos cardiovasculares futuros, demonstrando valor prognóstico superior.^7^ A base fisiológica é direta: órgãos vitais, incluindo o coração, o cérebro e os rins, são expostos à pressão dentro da aorta central, não à pressão amplificada encontrada no braço.^7^ Consequentemente, a pressão central serve como um indicador mais preciso do verdadeiro estresse hemodinâmico que esses órgãos sofrem.^7^ Há o reconhecimento da importância da pressão central para personalizar a terapia e evitar o tratamento excessivo no tratamento da hipertensão por meio de medidas de pressão arterial central.^8^ Além disso, a escolha da medicação anti-hipertensiva pode ser refinada considerando seus efeitos específicos na hemodinâmica central, como demonstrado no estudo CAFE. Diferentes classes de medicamentos podem ter efeitos divergentes na pressão central, mesmo quando seu efeito na pressão braquial é idêntico.^9^

Normalmente, os órgãos vitais empregam autorregulação, uma capacidade inata de preservar o fluxo sanguíneo constante, modificando a resistência arteriolar local em resposta às flutuações da PAM dentro de uma faixa de aproximadamente 50 a 150 mmHg.^10^ Em indivíduos com hipertensão crônica, a faixa autorregulatória pode ser necessária para manter a perfusão cerebral e renal adequada.^10^ No entanto, a meta ideal de PAM não é universal e deve ser adaptada à fisiologia específica do paciente.

Há uma diferença entre a dinâmica pulsátil da pressão aórtica e a medida integrada da pressão arterial média. A pressão aórtica fornece uma análise de alta fidelidade da complexa interação entre o coração e o sistema arterial, oferecendo insights sobre a função cardíaca e as propriedades vasculares. A PAM fornece um valor único e robusto que representa a força motriz líquida para a perfusão de órgãos. A compreensão das distintas funções fisiológicas e aplicações clínicas da pressão arterial aórtica e média é fundamental para o diagnóstico e o tratamento precisos de uma ampla gama de doenças cardiovasculares. Esses conceitos são complementares e visam integrar múltiplas camadas de informação para um atendimento mais personalizado ao paciente.
