# Supplementary material for: Medidas do Manguito Periférico para Estimativa da Pressão Aórtica Média
Source: Arq Bras Cardiol. 2025 Oct 14;122(9):e20250543. [Article in Portuguese] doi: 10.36660/abc.20250543 (PMC12674837; doi:10.36660/abc.20250543)
Supplement: Translation

# Translation: Peripheral Cuff Measurements for the Estimation of Mean Aortic Pressure

**DOI:** 10.36660/abc.20250543i

**Authors:** Weimar Kunz Sebba Barroso, Sayuri Inuzuka

**Affiliations:** 1 Goiânia GO Brazil Unidade de Hipertensão Arterial - Faculdade de Medicina - Universidade Federal de Goiás, Goiânia, GO – Brazil

**Keywords:** Blood Pressure, Measurements

For more than a century, the simple, office-based method of measuring blood pressure in the upper arm with a sphygmomanometer has remained the established standard for its measurement and management. Accordingly to hemodynamic principles, the primary function of the cardiovascular system is to ensure continuous perfusion of vital organs and tissues.^1^ The cardiac function and vascular dynamics generate the force known as arterial pressure.^1^ Aortic pressure describes the dynamic, pulsatile force exerted within the body's largest artery, reflecting the beat-to-beat interaction between the heart and the arterial tree.^1^ In contrast, mean arterial pressure (MAP) represents the time-averaged pressure that serves as the steady driving force for systemic blood flow.^2^ A comprehensive grasp of their core distinctions, underlying physiological mechanisms, and clinical significance is crucial for the proper diagnosis and treatment of a broad spectrum of cardiovascular diseases.

MAP is the average pressure that ensures a steady, continuous flow of blood to your body's tissues and organs.^2^ This constant perfusion is essential for delivering the oxygen cells need to function properly.^2^ The clinical concern is that to perfuse vital organs, there is a requirement of a minimum MAP of 60mmHg.^3^ If MAP drops below 60mmHg for an extended period, it may result in manifestations such as ischemia and infarction.^3^ There are several formulas for MAP calculation, and Gauer's formula was the first suggested.^2^


MAP=DP+1/3(SP-DP)or MAP=DP+1/3(PP)

Where DP is the diastolic blood pressure, SP is the systolic blood pressure, and PP is the pulse pressure. Aortic pressure is a dynamic pulsatile waveform that provides information about the quality of each cardiac cycle, which reflects the force of contraction, the integrity of the aortic valve, and the properties of the arterial tree.^1^ MAP is a steady-state, calculated value that resumes the average result of these cycles, representing the driving force for systemic perfusion.^4^

The authors of the study^5^ suggest a better formula for the MAP calculation (MAP=DBP+[0.33+(0.16−0.00107×PP)+(−0.06+0.000773×HR)]×PP). This formula uses data obtained by a conventional sphygmomanometer. The data obtained from this formula were compared with the intra-aortic pressure measured with coronary angiography. Through the years, significant effort has been dedicated to developing technologies that estimate the central pressure waveform non-invasively from a peripheral measurement site.^6^ The applanation tonometry is the most widely validated technique for non-invasively assessing the central pressure waveform, and more recent technological advancements have integrated waveform analysis into oscillometric blood pressure devices through device-specific algorithms that are used to derive and estimate the central aortic pressure waveform from the brachial waveform.^6^

The predictive value of central pressure has been widely investigated in cohorts in a large number of patients, most of them using tonometry-based techniques, which reported that central pressure was independently to future cardiovascular events, demonstrating superior prognostic value.^7^ The physiological basis is direct: vital organs, including the heart, brain, and kidneys, are exposed to the pressure within the central aorta, not the amplified pressure found in the arm.^7^ Consequently, central pressure serves as a more accurate indicator of the true hemodynamic stress these organs endure.^7^ There is the recognition of the importance of the central pressure to personalize therapy and avoid overtreatment in hypertension management using central blood pressure measurements.^8^ Furthermore, the choice of antihypertensive medication can be refined by considering its specific effects on central hemodynamics as demonstrated in the CAFE study. Different drug classes can have divergent effects on central pressure even when their effect on brachial pressure is identical.^9^

Typically, vital organs employ autoregulation, an innate capacity to preserve constant blood flow by modifying local arteriolar resistance in response to MAP fluctuations within a range of approximately 50 to 150mmHg.^10^ In individuals with chronic hypertension, the autoregulatory range may be necessary to maintain adequate cerebral and renal perfusion.^10^ Nevertheless, the ideal MAP target is not universal and should be adapted to the patient's specific physiology.

There is a difference between the pulsatile dynamics of aortic pressure and the integrated measure of mean arterial pressure. Aortic pressure provides a high-fidelity analysis of the complex interaction between the heart and the arterial system, offering insights into cardiac function and vascular properties. MAP provides a single, robust value representing the net driving force for organ perfusion. An appreciation for the distinct physiological functions and clinical applications of aortic and mean arterial pressure is critical for accurately diagnosing and managing a wide array of cardiovascular disorders. These concepts are complementary and aim to integrate multiple layers of information for more personalized patient care.
